# A critical assessment of monitoring practices, patient deterioration, and alarm fatigue on inpatient wards: a review

**DOI:** 10.1186/1754-9493-8-29

**Published:** 2014-06-27

**Authors:** J Paul Curry, Carla R Jungquist

**Affiliations:** 1 UCLA Department of Anesthesiology, Hoag Memorial Hospital Presbyterian, One Hoag Drive, 92663 Newport Beach, CA, USA; 2 University of Buffalo, Wende Hall Rm 314, 3435 Main Street, 14214-8013 Buffalo, NY, USA

**Keywords:** Alarm threshold values, Arousal failure, Central sleep apnea, CO_2_ narcosis, Continuous pulse oximetry, Dartmouth patient surveillance system, Functional residual capacity, General care floors, Obstructive sleep apnea, Oxygen supplementation, Oxyhemoglobin dissociation curve, Patterns of respiratory dysfunction, Rapidly evolving clinical cascades

## Abstract

Approximately forty million surgeries take place annually in the United States, many of them requiring overnight or lengthier post operative stays in the over five thousand hospitals that comprise our acute healthcare system. Leading up to this Century, it was common for most hospitalized patients and their families to believe that being surrounded by well-trained nurses and physicians assured their safety. That bubble burst with the Institute of Medicine’s 1999 report: To Err Is Human, followed closely by its 2001 report: Crossing the Quality Chasm. This review article discusses unexpected, potentially lethal respiratory complications known for being difficult to detect early, especially in postoperative patients recovering on hospital general care floors (GCF). We have designed our physiologic explanations and simplified cognitive framework to give our front line clinical nurses a thorough, easy-to-recall understanding of just how these events evolve, and how to detect them early when most amenable to treatment. Our review will also discuss currently available practices in general care floor monitoring that can both improve patient safety and significantly reduce monitor associated alarm fatigue.

## Introduction

Rapidly Evolving Clinical Cascades (RECC) are unexpected, often deadly adverse clinical events seen commonly in hospitalized patients. These events can manifest subtly at first or be totally disguised by sleep while clinical deterioration evolves through one of three distinct patterns of respiratory dysfunction. Because two of these patterns are associated with opioid administration, they are more likely to be found in postoperative patient populations where acute pain is routinely addressed and continuous monitoring is an exception rather than a rule [1]. While not every day occurrences, these events present in most hospitals and are associated with high mortality and morbidity because postoperative general care floor (GCF) workflow is not designed optimally for allowing clinical nurses to detect these events early [2,3]. Nor are our front line nurses specifically trained how to recognize these events at their inception, which leads to unintentional delays and more advanced deterioration [4]. When rapid response teams and in-house intensivist/hospitalist personnel are summoned and do address these problems, often the opportunity for achieving an optimal outcome has passed. More commonly, these patients are transferred in crisis to Intensive Care Units (ICU), leaving disrupted general care floors’ (GCF) nursing staffs with missed opportunities to learn from these complications. This review will discuss these kinds of clinical deterioration, the commonalities and differences in their physiologies, and the currently available strategies most capable of supporting early detection. Early recognition has been identified as the primary determinant of the success of intervention [3].

Much of our impaired ability to detect RECC early has stemmed from our dependence on monitoring strategies that are capable only of providing warning much later in the processes of deterioration [4,5]. Current general care floor (GCF) monitoring is often limited to isolated spot checks that include physiologic parameters such as the patient’s heart rate, respiratory rate, temperature, and the brief observations that come from an array of clinical and non clinical visits, all separated by significant time spans where no monitoring occurs. This kind of surveillance is typically done every 4 hours, which leaves patients unmonitored 96% of their total time spent on the GCF [6]. Some facilities provide patients with additional protection using continuous electronic monitoring (e.g. continuous pulse oximetry), but rarely are all GCF patients simultaneously afforded this level of surveillance [7]. When continuous monitoring is used, most of the chosen numeric values that must be breached to trigger its alarms (called alarm threshold values (ATV)) are physiologically extreme, e.g. heart rates > 130/min or < 50/min, respiratory rates > 30/min or < 8/min. This assures high statistical specificity, meaning few false positives with regard to something significant being wrong. It likewise assures less direct resource waste that otherwise would be incurred from having to respond to frequent false alarms. However, these extreme ATV can determine when our clinical nurses first take notice of the potential problem. Unfortunately, the time lost waiting for these highly specific alarm breaches often condemn patients to more advanced stages of clinical deterioration where correction now becomes emergent and much more costly in terms of resource utilization, morbidity and mortality [3]. In years past, the consequences comprising this additional cost were shifted to the traditional payers and fully remunerated. But that has changed, now with the cost of many unexpected complications deemed the responsibility of the institution where they’ve occurred. Longitudinal studies have demonstrated as well that any postoperative complication occurring within 30 days of surgery, no matter how trivial, is significantly more important than preoperative patient risk and intraoperative factors combined regarding associated long term reductions in survival regardless initial full recoveries [8,9].

The use of numeric thresholds derived from expert consensus has been practiced for decades but became most popular in the 1980s with the formalization of Threshold Decision Making [10] It was expert consensus, not clinical trial, that made way for a variety of physiologic threshold values to be selected to comprise the definition of sepsis in 1991, again in 2003, and reaffirmed in 2012 [11-13]. In 2014, this practice is now being highly scrutinized because of recent expert public disclosure announcing the near total lack of therapeutic progress made in the number one cause of hospital death - sepsis [14]. Many of its defining threshold values are now accepted as overly sensitive and too nonspecific to be clinically useful [15]. Several of the experts originally responsible for these threshold adaptations have come forward today with candor and courage, expressing their concerns that no gains in sepsis outcomes can be claimed other than improvements in its supportive care [14], while other experts point to the lack of large trial reproducibility as being this methodology’s largest collateral disappointment [16,17].

Yet, not all physiologic threshold values used in hospital patient care today have undesirable Receiver Operating Characteristics that render them useless. One example would be the 90% SpO_2_ (blood oxygen saturation) alarm threshold value (ATV) commonly used with continuous pulse oximeters in operating rooms, PACUs, and Critical Care Units, where patients often require ventilator support. This ATV makes great sense used in these settings where conditions like sudden endotracheal tube misplacement, displacement, or anesthesia influences exponentially increase the risk for sudden airway loss and abrupt respiratory arrest, all in the presence of experts focused on eliminating the threat to each individual patient at risk. Here, the alarm sounds in ample time for an attending anesthesiologist or skilled intensivist to take corrective action. But with the migration of continuous pulse oximetry to hospital general care floors in 1999, where continuous oximetry signals from whole populations of patients would be seen and heard at central nursing stations, the selection of this 90% SPO_2_ ATV quickly became a work place liability. False alarms abounded from unanticipated motion artifacts created by more mobile patient populations and rudimentary noise reduction algorithms embedded in the monitors themselves. True alarms abounded from a then yet to be appreciated high prevalence of self correcting, episodic apneas that recurred during patient sleep, arguably worsened with opioid use in post operative patient populations [7,18,19]. Regardless, well over a decade prior to alarm fatigue reduction strategies now required of hospitals in 2014 as a Joint Commission National Patient Safety Goal, GCF clinical nurses at the turn of this century struggled to maintain their professional composure when ordered to monitor their post surgical patients with these continuous pulse oximetry systems. The alarm clamor alone caused many to abandon the systems entirely [7]. Others created ‘expert’ consensus based patient selection processes designed to include only those patients thought to be at higher risk. Called condition monitoring, this strategy was used on identifiable conditions, e.g. diagnosed obstructive sleep apnea, but the outcomes documented failure from a lack of sensitivity [20]. Interestingly, no institution on record until 2007 tried tackling this ‘excessive alarming’ problem attributed to continuous pulse oximetry by simply adjusting its ATV [7]. (Instead of alarming at an oxygen saturation (SPO_2_) breach of 90%, one institution ‘increased’ its alarm threshold value (ATV) downward to 80% as will be discussed in detail later in this review). Regardless excellent outcomes from that one institution, the majority of hospitals utilizing continuous pulse oximetry on their general care floors today still select alarm threshold values set at or very close to 90%.

A couple of appropriate questions to ask now would be: Is there one best alarm threshold value (ATV) for continuous pulse oximetry monitoring on general care floors that we should be using to follow our patients, and should we be following all GCF patients simultaneously? The answers we provide in this review are simple, but the explanations that support them will be evidence based and founded on thorough physiologic understandings of the three Rapidly Evolving Clinical Cascade Patterns of Respiratory Dysfunction that frequent general care floors (GCF). It’s the latter that will give you lasting insight into exactly how to best detect these adverse events early where full recovery can be most expected.

### How is ventilation anatomy and physiology linked to our Three RECC pattern types?

We will begin our discussion by taking a moment to recall some basic ventilation anatomy and physiology. This is specifically designed to provide you with a simplified framework from which to easily remember the likenesses and differences of our three RECC pattern types. We start with a virtual anatomic structure called Functional Residual Capacity (FRC) that provides each of us with respiratory homeostasis. When RECC respiratory dysfunction does occur, it can be understood in terms of the particular changes that are taking place in this virtual FRC space. This can be useful when needing to process and communicate helpful information when directly dealing with early onsets of these events.

**Figure 1:**
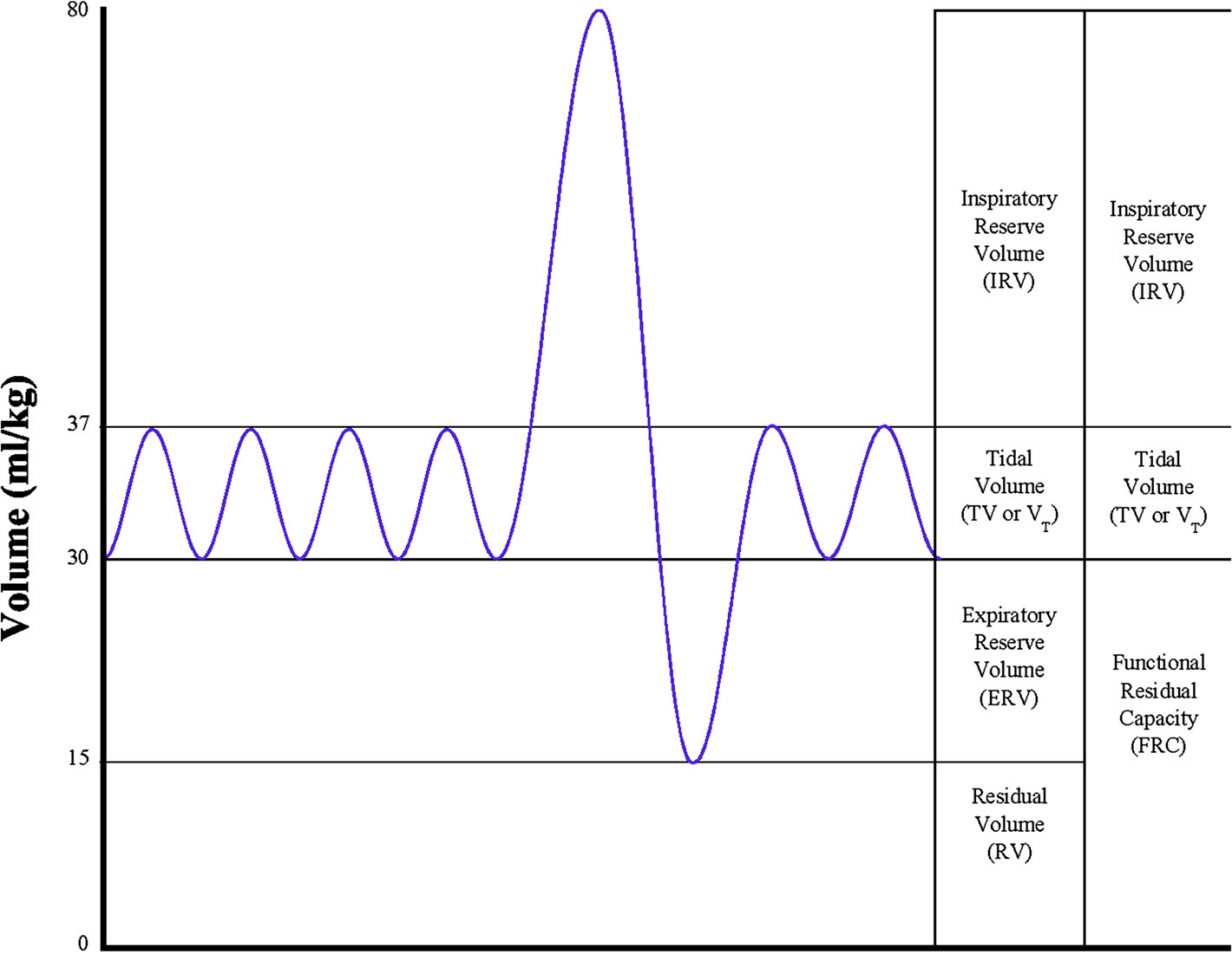
Lung capacities and volumes.

Functional Residual Capacity (FRC) depicted above in Figure 1 is an important, albeit virtual anatomic structure whose job is to continually provide our bodies any additionally needed oxygen beyond that being delivered within our moment to moment tidal volumes, so to maintain the stability our arterial oxygen content. It functions largely as an oxygen reservoir, playing a vitally important role as a necessary and constant contributor to our respiratory physiology, maintaining our generally stable arterial oxygen saturations. FRC is a combination of two real and separate anatomic volumes called Expiratory Reserve Volume and Residual Volume, but is more easily remembered as the lung air left over after normal exhalation. Our lungs hold approximately 6 L of air for men and less than 5 L for women at full capacity, achieved only during deepest inspiration. But on average our lungs normally operate at rest with our taking in tidal volume (V_T_) breaths of 500 ml ‘atop’ the FRC. Exhalation occurs approximately 16 times a minute leaving on average 2 L of air behind. Without our FRC, the tidal volumes we depend on to ‘freshen’ our FRC would only be capable of introducing oxygen into our circulations during a small portion of our ventilatory cycles. The FRC adds a comfortable cushion, allowing for continual restocking of oxygen desaturated blood that recurs reliably (to a point) even when lungs are partially damaged or breathing stops over short time intervals, like when consciously holding our breaths or with short episodic airway losses. Unfortunately, more prolonged apneas can deplete this FRC reservoir regardless how robust it might be under normal circumstances, which is germane to our coming discussions. But our primary reason for discussing FRC here is that all three RECC Patterns of Respiratory Dysfunction can be thought to have their indiviually distinct pathologic influences on it.

Respiratory dysfunction that involves acute progressive hypoxemia is a complex subject, comprising a myriad of supply and demand contributors other than FRC alteration. But our simplified FRC associations can be helpful tools to assist any clinical nurse when facing the possibilities of early RECC and calling for expert clinical backup. This should translate to providing patients their best chances to avoid needless complications due to delayed detection and intervention. Our RECC Type I pattern disrupts the FRC through a process of ‘*replacement*’ , while our RECC Type II pattern does it through a process of ‘*substitution*’ , and our RECC Type III pattern through a process we call ‘*bedside larceny*’. The process details will be explained next as we tackle each RECC pattern type in its numeric order.

### RECC Type I pattern of respiratory dysfunction

A healthy male who had just undergone elective surgery develops shortness of breath that’s noticed by his family who express concern to the nurse. The nurse, citing a normal oxygen saturation reading on his oximeter, reassures the family that the monitor indicates he’s okay. Eventually his respiratory rate does rise to a critical value, but by this time it’s too late to effectively respond to his rapidly deteriorating clinical condition and the patient, with sepsis, dies.

**Figure 2:**
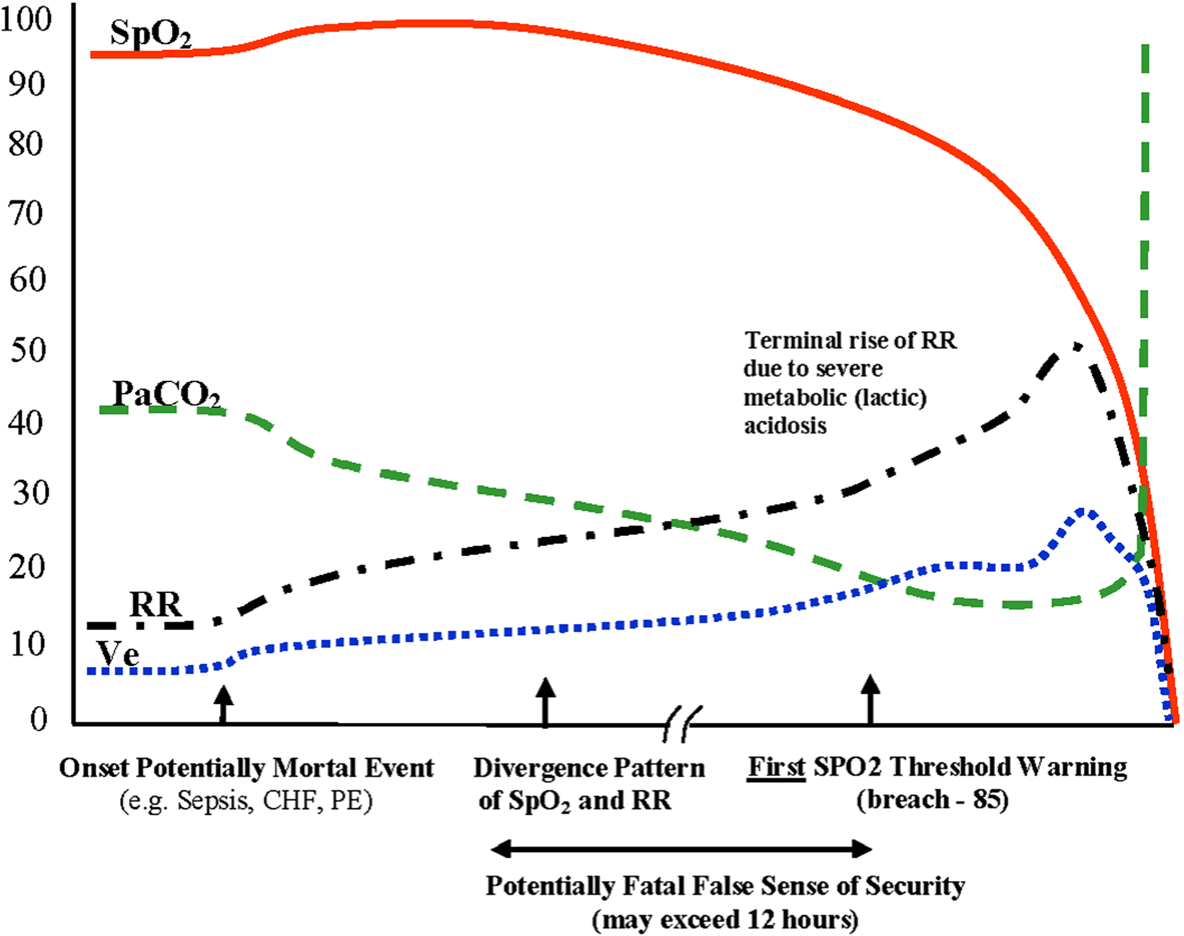
**RECC Type I pattern of respiratory dysfunction.** Details - SPO_2_: oxygen saturation; PaCO_2_: arterial carbon dioxide tension; PACO_2_: alveolar carbon dioxide tension; P-50: oxygen tension where hemoglobin is 50% saturated; Ve: minute ventilation; RR: respiratory rate.

This pattern of hyperventilation compensated respiratory distress reflects a clinically evolving process associated with microcirculatory failure induced by familiar RECC conditions like sepsis, congestive heart failure (CHF), aspiration, and pulmonary embolism (PE). It’s the most common pattern of our three, with prevalence for respiratory complications reaching as high as 2.7% in some postoperative populations [21]. Let’s first examine how the onsets of our four RECC examples above disrupt the FRC and its ability to stabilize oxygen saturation. The pattern unfolds with processes that begin to replace healthy lung (FRC) immediately. With CHF, water does the replacing. In sepsis, it’s pus (inflammatory factors). It’s gastric and bowel content with eventual pus in cases of aspiration, and with pulmonary embolism, portions of the FRC are replaced immediately (converted to dead space). Identifying the correct replacement process early, before a critical mass of lung is irreversibly harmed, becomes essential for optimal recovery.The Type I pattern generally begins with subtle hyperventilation and a persisting respiratory alkalosis (RA), regardless subsequent progressive increases in anion gap and lactic acid levels. This initial stage occurs well before the development of dominant metabolic acidosis (MA), which is usually associated with its later, and very late terminal stages. These progressive pattern phases (initially isolated RA followed by mixed RA and MA, in turn followed by dominant MA) comprise the typical progression seen (Illustrated in Figure 2 below).

The subtle early signs are easily overlooked. They are usually accompanied with complaints of mild dyspnea if patients are able to articulate their symptoms, and are often mistaken for anxiety. Nurses and physicians have on occasion been willing to discount these harbingers, even more so today than back in the pre-oximetry era, because now with spot oximetry checks so often available, patients may begin complaining but their SPO_2_ values are seen as remaining ‘normal’. What can be forgotten in the hectic, sometimes chaotic workflow of general care nursing is that normal appearing saturations in the mid to high 90s% can be misinterpreted as indicating respiratory stability when this isn’t the case. Any patient can maintain ‘good’ saturations early while advancing toward significant clinical deterioration because of the initial compensatory hyperventilation seen with Type I events. The patient’s actual unfavorably declining PaO_2_ changes [22] remain concealed because of this compensatory adaptation [23]. Respiratory alkalosis from hyperventilation and its affect on hemoglobin affinity hold saturations initially stable in early crisis. Our compensatory actions will deliver oxygen first and foremost when the respiratory system is challenged. Only later in the process does hydrogen ion stability, conformational changes of the hemoglobin molecule, and ultimately a precipitous fall in oxygen saturation (SPO_2_) combine to produce a resounding state of total respiratory collapse. Unfortunately, this later presentation can be when we first begin to realize our patients are in serious trouble, when it’s difficult to miss but much more difficult to reverse, with death likely to follow quickly. We will explore the Oxyhemoglobin Dissociation Curve (ODC) more thoroughly later with our review of the RECC Type II Pattern of Respiratory Dysfunction.

Any complaints or signs of dyspnea need to always be carefully evaluated at their onset. Unfortunately, this isn’t always the case. We have seen more than a few examples of very high respiratory rates (≥ 30/min) triggering rapid response team activations [24,25], and likewise triggering these patients’ first detailed evaluations, both late and sadly most often found in the non survivors when examined retrospectively [26].

While mildly elevated respiratory rates are known to be nonspecific markers for respiratory distress, when they are discounted until extreme respiratory rates begin to appear, they change into highly specific markers just like high lactate levels [27], but for the much later RECC Type I pattern manifestations of severe metabolic acidosis. Here these respiratory rates are best considered markers of severity and diagnostic delay [28], rather than useful warnings of early instability. Several studies have shown nursing spot checked respiratory rate recordings to be significantly unreliable [29], but automated, reliable continuous respiratory rate analysis is now available with many of today’s continuous pulse oximetry systems. Also available is a reliable, continuous non-invasive minute ventilation monitor that is capable of quantifying the ventilatory changes seen in our Type I pattern, and in theory could be combined with continuous pulse oximetry to provide the most reliable real time information needed for early detection of all Type I RECC events. However, no outcome trials have yet tested this logical assumption.

Until such trials do prove this to be of value to our clinical nurses, we will have to rely on education and learning from our mistakes to prevent delays in Type I detection. What is not unusual to find in practice is our nurses being asked to administer low flow supplemental oxygen to mildly dyspneic patients until a physician can come by to evaluate. If that physician’s evaluation is delayed, it isn’t unusual for standing orders to advance this supplemental oxygen, increasing it in incremental adjustments to maintain a specified SPO_2_ value. This adjustment process will continue to conceal the advancing pathologic FRC *replacement* changes, during which time the patient’s condition inadvertently further deteriorates. These iterative increases in supplemental oxygen conceal and delay accurate assessment by matching the dynamically deteriorating FRC *replacement* (injury) process with its own dynamic concealing process. Here oxygen can be harmful. The oximeter and its SpO_2_ values are erroneously reassuring until quite late when the Type I pattern has already turned deadly. These ‘supportive’ tactics, rather than the immediate workups and aggressive treatments these situations call for, become causative! The delays determine the poor outcomes, but these associations are unfortunately rarely appreciated. Rapid Response steps in when critical thresholds are breached and patients generally get whisked away to higher levels of care, considered often as victims of expected perioperative risk. Remember, supplemental oxygen should only be used in tandem with an aggressive search for a possible underlying RECC cause. There is no optimal oximetry alarm threshold value that is capable of warning us early that we are facing a RECC Type I Pattern of Respiratory Dysfunction. This is left to nursing judgment alone.

### RECC Type II pattern of respiratory dysfunction

A healthy female who is receiving routine post–op nasal oxygen has been up all night complaining of severe post-op pain, but is now finally asleep after yet another dose of IV opioid. The nurse, noticing on rounds the patient’s oxygen saturation is ‘perfect’ on the monitor, decides not to awaken her. She is found dead in bed 4 hours later.

**Figure 3:**
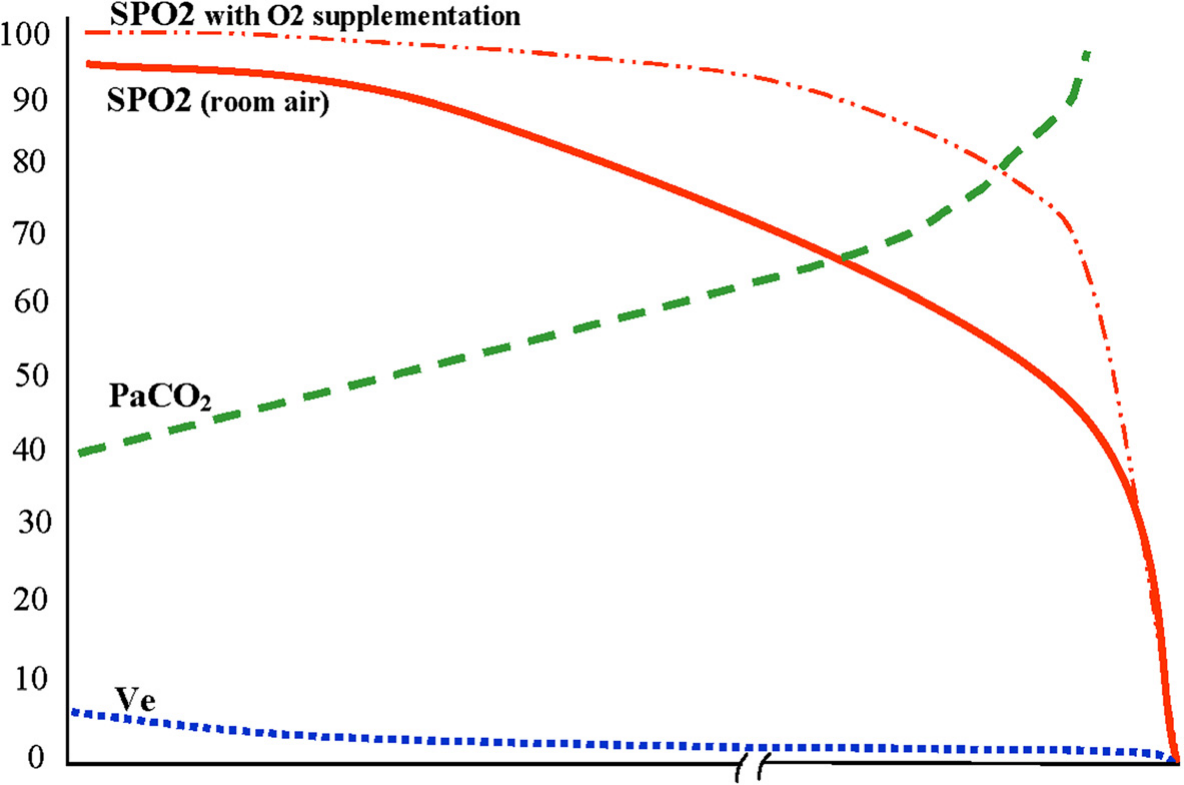
**RECC Type II pattern of respiratory dysfunction (CO**_**2 **_**Narcosis).**

Since the 1950s, nurses and physicians in training have learned that opioids produce death through this singular path involving progressive unidirectional hypoventilation [30]. The synergy of opioids and a rising PaCO_2_ contributes to central depression of our ventilatory drive, ultimately leading to ‘CO_2_ Narcosis’, an unstable condition that if left unchecked will lead to respiratory arrest. Opioid associated events aren’t unusual in hospitals today. Experts speculate that up to a third of all code blue arrests in hospitals could result from opioid induced respiratory depression [31], and naloxone is administered as an antidote for opioid associated events in 0.2-0.7 of patients receiving them postoperatively [32,33]. One estimate has these representing 20,000 of our nation’s patients annually with one tenth suffering significant opioid related injuries that include death [34]. These events are always catastrophic, devastating to patients, patient families, and all clinicians involved. Yet in spite of all this incentive to improve, we have not made meaningful progress in protecting our patients from these events for a host of reasons, one major contributor being the increased emphasis on optimal postoperative pain management by centers that govern reimbursement [6] (Figure 3).

The RECC Type II pattern above exhibits a significant diminution in both inspiration and expiration airflow that results in significant quantitative changes in the amounts of gases normally occupying lung. Because these partial pressure changes equilibrate immediately with arterial blood minus an age predictable A-a gradient, once extreme levels are reached they can cause biochemical dysfunction at all end organ sites, most importantly the brain. Specifically, CO_2_ continues to mount in the blood (PaCO_2_) and lung (P_A_CO_2_), unable to be cleared because of the opioid induced ventilatory depression. When levels of PaCO_2_ have climbed to ‘narcotizing’ thresholds in the 70 mmHg range, carbon dioxide’s own respiratory depressive effect begins to assert, combining with the opioid’s effect to advance and accelerate a covert respiratory failure and severe respiratory acidosis. On the postoperative GCF, these respiratory failures are often easily confused with blissful sleep while extraordinarily high PaCO_2_ levels continue to mount. The Type II pattern is frequently discovered only by accident when unsuccessful attempts are made to arouse these patients.

Some patients are at very high risk for postoperative hypoventilation even when given ‘normal’ doses of sedatives and opioids. Patients with congenital central hypoventilation syndrome [35] can be completely asymptomatic while awake, yet despite their normal daytime PaCO_2_, exhibit profound hypoventilation responses to sedation and opioids when asleep. Others at risk include patients with obesity hypoventilation syndrome [36], chest wall deformities, polio sequelae, advanced COPD [37], and severe hypothyroidism [38].

Our current practice for detecting opioid induced respiratory depression is to monitor the respiratory rate, and while some studies have shown that respiratory rate reductions provide a useful indication of ventilatory depression in some patients [39,40], there’s ample evidence to suggest that it’s not that simple. Several studies have shown opioid and sedative induced respiratory depression to be associated with reductions in tidal volume and more variable patterns of breathing [41-43]. In fact, hypoventilation produced by some benzodiazepines may primarily reduce tidal volumes with accompanying increases in respiratory rate [44]. In obese patients, or others with narrow, semi-patulous upper airways, tidal volumes may be even further reduced through increases in opioid associated upper airway resistance [45,46], suggesting that any relative reductions in rate and/or tidal volume are likely to be highly variable depending on both patient and drug-related factors. So, relying on threshold respiratory rate monitoring as our only surrogate marker for opioid induced respiratory depression may only be providing a false sense of security.

The addition of continuous pulse oximetry surveillance to intermittent or continuous respiratory rate monitoring can be very helpful when detecting Type II RECC events unless patients are simultaneously receiving supplemental oxygen [47]. When hypoventilation progresses while certain flow rates of supplemental oxygen are being provided, the escalating CO_2_ retention process can remain hidden from pulse oximetry monitoring until very late when lethal levels of CO_2_ have already accumulated [48-50]. This masking effect from the supplemental oxygen may result in deadly detection delays much like what can happen with the Type I pattern, but for an entirely different reason. Without supplemental O_2_, the expected Fraction of Inspired Oxygen (FIO_2_) contained in the air we entrain into our lungs with each breath we take is immediately diluted into much leaner partial pressures because of how the Type II RECC process influences the FRC. Where Type I RECC processes can be thought to *‘replace’* the FRC itself, the Type II RECC process *‘substitutes’* one gas’s progressively rising partial pressures (P_A_CO_2_) in healthy lung for the partial pressures of the other gases remaining from inhaled room air (N_2_, H_2_O, and O_2_). It’s a pathologic dilution process where all the partial pressures in the lung, when added together, will equal the expected atmospheric pressure with only one (P_A_CO_2_) increasing. The trapped, climbing P_A_CO_2_ competes for the same ‘FRC’ space as the fixed partial pressure components of air, diminishing the latter, most importantly the P_A_O_2_. The alveolar gas equation:

$$\left[P_{A}0_{2}=F_{I}O_{2}\left(P_{\text{ATM}‒}PH_{2}O\right)‒P\text{aC}O_{2}/\text{RQ}\right]$$

can approximate what these expected partial pressure values of the diluted (*substituted*) alveolar oxygen would be in people with lungs that function normally. For example, at sea level breathing room air with a normal PaCO_2_ of 40 mmHg, we can expect the P_A_O_2_ to be 99.7 mmHg. If the PaCO_2_ rises to 50 mmHg, the P_A_O_2_ falls to 87.2 mmHg, 60 mmHg PaCO_2_ = 74.7 mmHg P_A_O_2_, and 70 mmHg PaCO_2_ (CO_2_ Narcosis) = 62.2 mmHg P_A_O_2_. Few clinicians are able to precisely state off hand what early SPO_2_ oximetry changes are to be expected if sleeping patients receiving parenteral opioids are being monitored with continuous pulse oximetry and begin progressively retaining known elevations of PaCO_2_ from a RECC Type II process. This holds especially true for patients with otherwise normal lungs who are simultaneously receiving supplemental oxygen. We will now connect a series of basic physiologic calculations like those above to allow you to understand just how this can be worked out accurately. This will likewise demonstrate the limitations to continuous pulse oximetry monitoring when surveilling for the Type II Pattern of Respiratory Dysfunction.

Assuming normal breathing patterns and pulmonary physiology, it isn’t difficult to reasonably estimate from the P_A_O_2_ what the PaO_2_ should be. Generally, the P_A_O_2_-PaO_2_ (A-a gradient) when breathing room air can be calculated simply by taking a patient’s age, adding 10 to this value, and dividing the sum by 4. So assuming an otherwise healthy 50 year old patient breathing room air has a P_A_O_2_ that rounds off to 100 mmHg, his PaO_2_ can be expected to approximate 85 mmHg (15 mmHg A-a gradient). And once we know the PaO_2_, we can estimate his blood oxygen saturation (SPO_2_) with reasonable accuracy, but only if we account for the variables that influence hemoglobin saturation. These variables are the parameters capable of shifting the Oxyhemoglobin Dissociation Curve (ODC) to the right or left, such as his concurrent arterial blood pH, PaCO_2_, background 2,3DPG (Diphosphoglycerate), and temperature. There could be hidden variables as well, unique to an individual patient that could skew any of these mathematical outcomes unexpectedly, but generally these calculations are valid and relatively precise. Automated mathematical models like HbO. Severinghaus and HbO.Dash are used today in blood gas laboratories to correlate reliable saturation values off any known PaO_2_, provided we accept certain assumed fixed values for some of the less important variables, yet account for the most important ones. The HbO.Dash model offers one large correction advantage by allowing us to factor a patient’s concurrent arterial blood pH and PaCO_2_ into its saturation determination. This provides a significant correction because it accounts for the Bohr effect, a major physiologic modifier that shifts the ODC increasingly rightward or left, the more acidotic or alkalotic a patient’s arterial blood might become. Respiratory acidosis (elevated PaCO_2_) and its rightward curve shifts can significantly decrease the expected saturation values on any given PaO_2_. Leftward shifts associated with respiratory alkalosis (diminished PaCO_2_) raise expected saturation values for any given PaO_2_, like that caused by the hyperventilatory respiratory alkalosis seen in early Type I RECC processes. The same ODC shifts hold true for metabolic acidosis and alkalosis, but it’s acute respiratory alkalosis that contributes significantly to concealing early Type I FRC ‘*replacement*’ processes from the oximeter by stabilizing SPO_2_ while the PaO_2_ continues to deteriorate. Both early Type I and Type II processes can likewise be concealed from oximeters when supplemental oxygen is delivered indiscriminately.

Progressively worsening respiratory acidosis can be assumed from any rapidly evolving unidirectional hypoventilation, a biochemical consequence of the mounting CO_2_ retention. With these immediate onsets of respiratory acidosis, an arterial blood’s pH value in an otherwise healthy individual can be predicted directly from knowing the PaCO_2_ value and using a Henderson-Hasselbach equation calculator (pH = 6.1 + log(HCO_3_/(0.03 × PaCO_2_)), because the equation’s bicarbonate (HCO_3_) variable remains fixed acutely at 24 mEq/L. (It generally takes 3–5 days for our kidneys to begin compensating for any acute respiratory acidosis by preserving additional HCO_3_) Pulse oximeters measure saturation directly and don’t sort out which variables are at work altering the Oxyhemoglobin Dissociation Curve (ODC) or age related diffusion changes to make that SPO_2_ possible.

If patients on opioids progressively accumulate CO_2_ while breathing room air, the ‘*substitution*’ process will manifest immediately on continuous pulse oximetry monitors because the substitution and dilution of the original FIO_2_ entrained into the lungs by the retained CO_2_ translates immediately to substantial progressive reductions in PaO_2_ and its associated SPO_2_. It isn’t nearly so straightforward when supplemental oxygen is being delivered, especially when the route most commonly used to deliver it is through nasal cannulas. You will soon learn how and why supplemental oxygen delivery can be imprecise under the best of circumstances, which should improve your vigilance regarding monitoring and trouble shooting any potential Type II RECC events. We wish for you to fully appreciate the logic and relationships involved regarding surveilling for Type II events because they are arguably the easiest to miss should you not have the information we are about to share. Once we detail these precise physiologic relationships, we will leave you with a table that demonstrates for your future referencing the oxygen saturation changes you might reasonably expect to see (with caveat) from a variety of sleeping patient models experiencing progressive opioid induced Type II processes while simultaneously receiving a selection of possible supplemental oxygen flow rates.

Most continuous pulse oximetry monitors including those used on GCF, are set to alarm once the SPO_2_ value dips below 90% [1,7]. This particular GCF practice continues to be associated with alarm fatigue, alarm neglect, continuous monitoring exclusion, and poor opioid associated GCF outcomes [7,31,34]. Nor is it practiced universally in the United States. Dartmouth’s Hitchcock Medical Center has been deploying universal GCF pulse oximetry monitoring with their alarm thresholds set at 80% since 2007, and boasts no opioid associated deaths or anoxic brain injuries from the time of this inception [7,51]. Nevertheless, 90% thresholds still account for the majority of GCF practices today for reasons that have never been clarified by clinical trial, but that will be discussed once we have covered all three Patterns of Respiratory Dysfunction. If we wish to be clinically competent to manage patients receiving parenteral (and neuraxial) opioids on GCF, one of our skill sets should be the capability of reliably detecting opioid associated CO_2_ Narcosis (the RECC Type II pattern) in its earliest stage when PaCO_2_ approximates 70 mmHg, where it rarely causes harm and can be easily corrected. To do this, we need at minimum to be aware of the fundamentals of opioid induced respiratory dysfunction, including an appreciation for its complexity. Let’s look again at our healthy 50 year old model with normal lungs, but now we will make him a PACU patient with you in charge. We will also compare him to two additional patients, healthy 30 and 75 year olds, to get you comfortable with the vagaries of this Type II respiratory dysfunction and opioid associated influences. (We will further assume that your hypothetical PACU is at sea level for those of you clever enough to insist on this amount of granularity).

All three of your hypothetical patients have gone through operations where there is minimal blood loss but significant pain. For our first vignette, assume each is about to leave the PACU on room air, all are comfortable from appropriately administered opioids, all easily aroused with saturations (SPO_2_) of 92% and breathing at 10-12/min. They have all been started on standard, demand only morphine PCAs, and will be monitored with continuous pulse oximetry once on the GCF. What might be your concerns? You certainly have done a nice job managing their pain, and there seems to be physiologic evidence that opioids are producing some respiratory depression along with high quality analgesia. Because these are otherwise healthy individuals, and you feel somewhat confident in the safety profiles of demand only PCAs, you shouldn’t feel a need to subject these patients to arterial sampling for blood gas analysis. What you might want most is reassurance that should these patients become progressively more narcotized to the point of CO_2_ Narcosis (PaCO_2_ approximating 70 mmHg), that this will be reliably detected and corrected. You also know how busy the GCF can get and that your GCF oximeters alarm once SPO_2_ values breach 90%, just like those in your PACU. Even though reports have indicated that severe hypercapnic acidosis is surprisingly well tolerated [52], if you are sensitive to the American Heart Association’s ACLS recommendations regarding severe acidosis being defined as arterial pH < 7.2 and that standard Critical Care Admission Criteria for many hospitals today includes arterial pH < 7.25, you might also like to know that your patients’ arterial pH stay above 7.25 (*which in uncomplicated respiratory acidosis is done by limiting the maximum PaCO*_*2*_*to no higher than 55 mmHg*). So accepting these provisos, let’s have a look at how and why our mathematical formulas and simulation models can project how safe we are capable of keeping these patients on the GCF using continuous pulse oximetry surveillance.

Your 30 year old patient breathing room air will have a P_A_O_2_-PaO_2_ (A-a gradient) of 10 mmHg, your 50 year old a 15 mmHg gradient, and your 75 year old a 21 mmHg gradient. You can’t calculate precisely what your P_A_O_2_ will be because you don’t know what your PaCO_2_ is, but you do know you prefer the PaCO_2_ to not exceed 55 mmHg because a PaCO_2_ 56 mmHg = arterial pH 7.25. So working backwards with the assumption that your 30 year old’s PaCO_2_ is 55 mmHg, his P_A_O_2_ calculates out to be 81 mmHg. Subtract his A-a gradient of 10 mmHg and his PaO_2_ will be 71 mmHg. This value correlates to a HbO.Dash SPO_2_ computation value of 91%, 1% beneath his current PACU SPO_2_ 92%, indicating at the moment a job well done since his PaCO_2_ must be somewhere beneath your 55 mmHg limit. But there’s not a lot of cushion. The HbO.Dash computation also tells you with appropriate recalibrations of his P_A_O_2_ that this patient’s PaO_2_ would need to drop to 67 mmHg to trigger a pulse oximeter’s alarm set for a 90% SPO_2_ breach. It further quantifies that any such breach would correlate with a PaCO_2_ of 58 mmHg that calculates to an arterial pH of 7.24, neither ideal but well beneath a PaCO_2_ of 70 mmHg where emergent, though relatively simple therapeutic interventions would be needed to bring ventilatory performance back in line.

Your 50 year old has a 15 mmHg A-a gradient, so working with the same assumptions, his PaO_2_ drops to 66 mmHg. This correlates to a HbO.Dash SPO_2_ value of 89%. Clearly, with his PACU SPO_2_ value of 92% on room air, his PaCO_2_ and pH must be well within acceptable ranges. Additionally, his computations show us that the 90% ATV pulse oximeter will begin alarming (SPO_2_ 89%) if his PaCO_2_ does climb to 55 mmHg. Your 75 year old has a 21 mmHg A-a gradient that would drop his PaO_2_ to 60 mmHg at a PaCO_2_ of 55 mmHg. This correlates to a HbO.Dash SPO_2_ value of 87%, leaving even more margin for safety regarding his arterial pH with his PACU SPO_2_ at 92%. In fact, the opioid respiratory depression we fictitiously assigned him doesn’t really align with his calculated clinically near normal PaCO_2_ of 46 mmHg and arterial pH of 7.34 that we worked out with multiple simulated HbO.Dash computation runs. But any PaCO_2_ higher than 50 mmHg would drop his SPO_2_ below 90%, causing the GCF continuous pulse oximeter to alarm needlessly, assuring unwanted nursing distraction should he choose to begin pushing his PCA demand button. Unfortunately, it would also assure little rest for him and unsafe GCF conditions for everyone because of alarm fatigue. One way to circumvent this issue would be to provide him with supplemental oxygen, as we are accustomed to doing. But as will be demonstrated, this decision comes at a steep price.

Back again to your 30 year old patient… Instead of breathing room air in the PACU, he’s put on 3 L/min O_2_ through nasal cannulas. Let’s assume that this raises his FIO_2_ from .21 to .30, a commonplace assumption (.21 + (.03 × O_2_L flow/min)) when using this kind of oxygen delivery system [53]. Now his A-a gradient must be adjusted to accommodate this approximate .1 FIO_2_ addition, and the new gradient is estimated by adding an additional 5–7 mmHg to his original A-a gradient for each added .1 FIO_2_ increase. Because the FIO_2_ is now .3, his calculated P_A_O_2_ (assuming a PaCO_2_ of 55 mmHg) will now be 145 mmHg. So we subtract from this *P*_A_O_2_, his (age + 10)/4 + the additional 7 mmHg A-a adjustment, and his PaO_2_ approximates 128 mmHg. This PaO_2_ correlates to a HbO.Dash SPO_2_ above 98%, meaning that at an arterial pH of 7.26 and PaCO_2_ of 55 mmHg, the pulse oximeter will not indicate any sign of trouble. At a PaCO_2_ of 70 mmHg (arterial pH of 7.16) his P_A_O_2_ calculates to 126.4 mmHg and his PaO_2_ with A-a adjustments approximates 109 mmHg. This yields a 95% SPO_2_, a value that rarely earns more than cursory notice, yet now your patient is in serious trouble while probably appearing to be sleeping blissfully in no distress at all! Your 50 year old patient will have a near identical experience because with all the adjustments appropriate for his age, at a PaCO_2_ of 70 mmHg (arterial pH of 7.16) his PaO_2_ calculates to 104 mmHg with a HbO.Dash SPO_2_ value again of 95%! Your 75 year old doesn’t fair any better. At a PaCO_2_ of 70 mmHg (arterial pH of 7.16) his PaO_2_ calculates down to 98 mmHg with his A-a adjustments, but the HbO.Dash SPO_2_ remains in the 95% range, just a tad lower by fractions of a decimal point. Now let’s see how things might improve as we attempt to limit our ‘low’ flows of supplemental O_2_, starting at 1L/min.

1L/min O_2_ flows though nasal cannulas by convention will bring the FIO_2_ from .21 up to .24, although we’ll share a caveat regarding this in a moment. Assuming a FIO_2_ of .24 and a PaCO_2_ of 55 mmHg, the P_A_O_2_ calculates to 102.4 mmHg. Adjusting for the A-a gradient, your 30 year old will have a PaO_2_ of 89.4 mmHg and a HbO.Dash SPO_2_ of 95%, which as mentioned above will not normally draw a lot of attention unless the bedside nurse is very well informed about these basic science details. Your 50 year old will have a PaO_2_ of 84.4 mmHg at a PaCO_2_ of 55 mmHg, with a HbO.Dash SPO_2_ of 94%, and your 75 year old will have a PaO_2_ of 78.4 mmHg with a HbO.Dash SPO_2_ of 93%. Said another way, these patients may be comfortable and will probably not attract much clinical attention based on their SPO_2_, even though their respiratory acidoses (arterial pH 7.26) teeter at a generally well tolerated [52] moderately severe level. What happens if we drive the PaCO_2_ up to 70 mmHg where truly dangerous CO_2_ Narcosis comes into play? Here the P_A_O_2_ calculates to 83.6 mmHg. Adjusting for the A-a gradients, your 30 year old will have a PaO_2_ of 70.6 mmHg and a HbO.Dash SPO_2_ of 89%, which guarantees a 90% fail safe threshold oximeter alarm. Your 50 year old will have a PaO_2_ of 65.6 mmHg at a PaCO_2_ of 70 mmHg, with a HbO.Dash SPO_2_ of 87%, and your 75 year old will have a PaO_2_ of 59.6 mmHg with a HbO.Dash SPO_2_ of 83%-84%.

It appears that delivering 3L/min supplemental O_2_ flows through nasal cannulas is capable of leaving little to no clue on continuous pulse oximeters that lethal Type II patterns have evolved until quite late. With alarms set to sound at 90% SPO_2_ breaches, early CO_2_ Narcosis will have been missed. Nor is any significant SPO_2_ downward drifting trend immediately evident at this flow rate. On the other hand, if supplemental O_2_ must be used, containing it to 1L/min flow rates does allow detection of early CO_2_ Narcosis both by relying on the continuous pulse oximetry’s safety net 90% ATV and by a far more desirable nursing practice – direct observations of SPO_2_ trends drifting downward before any alarm sounds. Here, being aware and vigilant regarding these unexpected downward trends of SPO_2_ on ‘sleeping’ opioid managed patients can reliably give CO_2_ Narcosis away prior to any alarm and harm.

Unfortunately, another important issue must be addressed before leaving this subject. There can be striking variations with FIO_2_ when supplemental oxygen is being delivered through nasal cannulas. A patient’s actual FIO_2_ gain while receiving ‘low flow’ oxygen supplementation (traditionally 1–3 liters/min), can increase significantly if that patient, who normally pulls in robust tidal volumes suitable to age and size, now becomes narcotized to where the associated respiratory depression reduces tidal volume and minute ventilation to half its normal size. Flow rates and the actual percentage of oxygen entrained into patients’ lungs can be unpredictable and independent of one another unless specialized equipment like Venturi systems are used. Calculating accurate FIO_2_ with nasal cannulas requires knowing the patient’s minute ventilation and fraction of oxygen flow actually reaching the lung. A ‘rule of thumb’ shortcut suggesting for every iterative oxygen liter flow increase, that the FIO_2_ can be expected to climb .03 beyond the .21 in room air [53], is based on assumptions of average tidal volumes and ‘guestimates’ of actual entrained oxygen content. All of this can be wildly disparate from one patient to the next, especially when significant opioid induced respiratory depression is involved.

We’ll close this discussion with one last set of HbO.Dash computations, designed to test if early CO_2_ narcosis (PaCO_2_ approximates 70 mmHg) can be detected on an oximeter at an FIO_2_ of .27 (conventionally associated with 2L/min O_2_ flow rates through nasal cannulas). Your expected P_A_O_2_ at a PaCO_2_ of 70 mmHg will be 105 mmHg. Your 30 year old patient’s A-a gradient adjusts his PaO_2_ to 90 mmHg and a HbO.Dash SPO_2_ of 93%-94%, your 50 year old adjusts to a PaO_2_ of 85 mmHg and a HbO.Dash SPO_2_ of 93%, and your 75 year old adjusts to a PaO_2_ of 79 mmHg and a HbO.Dash SPO_2_ of 91%. No alarms sound from a 90% SPO_2_ threshold breach, but the SPO_2_ downward trend becomes quite evident, and can forewarn an astute bedside nurse that CO_2_ Narcosis should be ruled out. How do we rule this out? Simply go to these patients’ rooms, observe if they are ‘sleeping’ and if they are, try arousing them. Remember, these comfortably content ‘sleepers’ can be unresponsive. Unless we act to determine that they are arousable, we’ll never know for certain this is the case. And if it isn’t, this is the second way that supplemental oxygen can harm patients. It isn’t necessarily a bad practice to deliver small doses of supplemental oxygen to postoperative patients (1L and perhaps even 2L/min if the clinicians ordering it are concerned about tissue oxygenation and are cognizant of the SPO_2_ masking issues that arise from excessive flow deliveries), just potentially dangerous in environments where no one appreciates the physiologic relationships just discussed. Patients requiring supplemental oxygen for co morbid respiratory disease, who simultaneously are receiving parenteral (and/or neuraxial) opioids, present a more complex management challenge than can be covered in this review. Generally, these cases call for more prescriptive plans that often involve higher levels of care than that routinely provided on hospital general care floors.

To summarize, RECC Type II PUHD comprises first a progressive fall in minute ventilation due to declines in tidal volume and/or respiratory rate, both unpredictably variable. This induces a progressive rise in both PaCO_2_ and the CO_2_ retained by the FRC (P_A_CO_2_) that eventually *‘substitutes’* for its other essential gases, e.g. O_2_. When breathing room air, this *‘substitution’* process can be detected quite early using continuous pulse oximetry and either a safety net alarm threshold value (ATV) set at 90%, or astute observations made of downward SPO_2_ trends preceding any alarm breach and their likely association with rising sedation scores. (Some might correctly argue that our 75 yo patient model breathing room air actually showed our 90% SPO_2_ safety net threshold alarm to be too non specific, where false positive alarms would begin to sound in this particular instance without any danger actually existing). Providing supplemental oxygen at an FIO_2_ above .27 can conceal these early *‘substitution’* processes from continuous pulse oximetry, making detection of impending ‘CO_2_ Narcosis’ exceedingly difficult. These patients may progress to the point of stupor and even death if unfortunate enough to not have anyone attempting to arouse them. From a surveillance perspective, this is the one RECC pattern that can be detected using continuous, real-time CO_2_ monitors. However, important issues associated with choosing this strategy, excluding its significant additional cost, center on:

• Agreeing to a value that would define the alarm threshold best representing a legitimate clinical threat.

• Patient intolerance for the monitoring and sensors needed [7].

• How best to reverse the respiratory depression once detected, without neutralizing the just as important analgesia that has triggered the initial problem.

Optimal treatments for Type II events involve utilizing ventilatory pressure support, e.g., CPAP and/or BiPAP. Unfortunately, late detection of RECC Type II PUHD more often than not leads to full naloxone reversal tactics combined with either ventilatory pressure support or crash intubation, depending largely on how advanced the clinical deterioration, the perceived frailness of the patient, and the experience of the rescuer involved. Because CO_2_ doesn’t play a direct role in either RECC Type I or Type III patterns (*as we will learn next*), this monitoring strategy really is of limited use as a continuous, stand-alone first choice for our GCF where all three RECC patterns occur randomly. Real-time continuous respiratory rate monitoring can be quite specific, but is known to be unacceptably insensitive with far too many false negatives [29] as already discussed. Reliable continuous non-invasive minute ventilation is now available (Respiratory Motion Inc. *ExSpiron*™), but currently its sensors are quite expensive, making universal GCF surveillance at the moment impractical, although it will in theory yield very valuable information on all three RECC Types, especially if combined with continuous pulse oximetry. By providing accurate, real-time quantifications of tidal volume reductions associated with ongoing opioid management and SPO_2_, continuous minute ventilation should be able to help make supplemented oxygen FIO_2_ estimations far more reliable. In turn this would permit supplemental O_2_ delivery to be adjusted precisely to where it needs to be to assure that the SPO_2_ downward trends would always be detectable, likewise assuring reliable detection of early CO_2_ Narcosis using continuous pulse oximetry. Clinical trials will have to demonstrate these assumptions to be true.

Bedside nurses can be very helpful ‘monitors’ if knowledgeable, but require a working understanding of all three RECC patterns in order to be able to efficiently adjust their workflow to provide optimal safety, even when using one continuous electronic monitor like pulse oximetry [7,20,51]. Without any continuous monitoring, opioid associated adverse events continue to persist in postoperative populations [20,31-34], so from what we now know about RECC and two of our three coexisting pattern types, it’s difficult to argue a case for leaving patients unmonitored at all. Intermittent nursing checks, especially when opioids are involved, will always leave patients unobserved over 90% of their time on the GCF [6] regardless how enlightened the nurses might be. Many experts now argue soundly that healthcare providers should partner with at least one continuous electronic form of surveillance to make any GCF environment truly safe [20,54]. An argument for the most appropriate continuous monitoring surveillance will follow once we’ve mastered all three RECC patterns types, our last pattern, the RECC Type III Pattern of Respiratory Dysfunction next.

### RECC Type III pattern of respiratory dysfunction

An otherwise healthy male with unrecognized sleep apnea receives a post-operative opioid. His alarm sounds repeatedly but lasts only for about 30 seconds before it stops, only to repeat again and again. When the nurse awakens the patient he feels fine and is completely alert, asking for more pain medication, which the nurse gives in a normal dose. The nurse, suffering from alarm fatigue, stops responding to the same alarming. Later that night the patient is found dead in bed.

This is our third and final pattern, defined by its sentinel rapid airflow/SPO_2_ reductions commonly associated with obstructive sleep apnea (OSA) that then are followed by a precipitous, terminal SPO_2_ fall. Said another way, it is a sleep apnea event with no self rescue arousal component intervening (*arousal arrest*). Without our normally expected arousals, any sleep apnea event will terminate as a hypoxemic full respiratory arrest unless someone other than the patient is present to intervene. Cyclical airflow reductions and apneas associated with OSA allow both transient hypoxemia and transient elevations in PaCO_2_, the latter normally a reliable contributor to generating our arousal response. However, there are medications [45,46,55,56] and consequences of medical procedures [57] that may increase these CO_2_ arousal thresholds to levels so much higher that even the slightest additional arousal delay (e.g., from repetitive PCA opioid administrations) may allow critically low SPO_2_ levels to be reached. Likewise, conditions that encourage ongoing cycling hypoxemia [58], e.g. depleted oxygen reserves (venous and FRC), can have the same effect by permitting accelerated desaturations to reach these critical levels before even normal CO_2_ arousal generating thresholds are reached. Both conditions are prevalent on postoperative GCF where these Type III Patterns of Respiratory Dysfunction are known to occasionally occur [59-61].

While the actual general population prevalence of Obstructive Sleep Apnea (OSA) in America is estimated at 22% (approximately 80 million people with up to three-quarters having moderate to severe symptoms and remaining undiagnosed [62], the RECC Type III pattern is likely the most frightening and abrupt of our three pattern types. Its actual prevalence is muddled with potential crossover from both opioid associated Type II respiratory arrests, and from both Type II and Type III likely assigned incorrectly to unwitnessed cardiac arrest databases as mentioned in our discussion of RECC Type II patterns. Because of the sudden, and very often unwitnessed nature of these events, the term Dead-in-bed syndrome is sometimes used to describe them. Hospital Dead-in-bed prevalence can arise from any number of causes, e.g. those associated with diabetes are estimated at 2-6/100,000 patients [63], while as we’ve already mentioned, parenteral opioids in hospitals have been associated with 20,000 untoward annual events, 1/10th of which are known to involve serious sequelae that would include global anoxic brain injury and Dead-in-bed events. Both of these latter conditions can result from Type II and Type III events.

**Figure 4:**
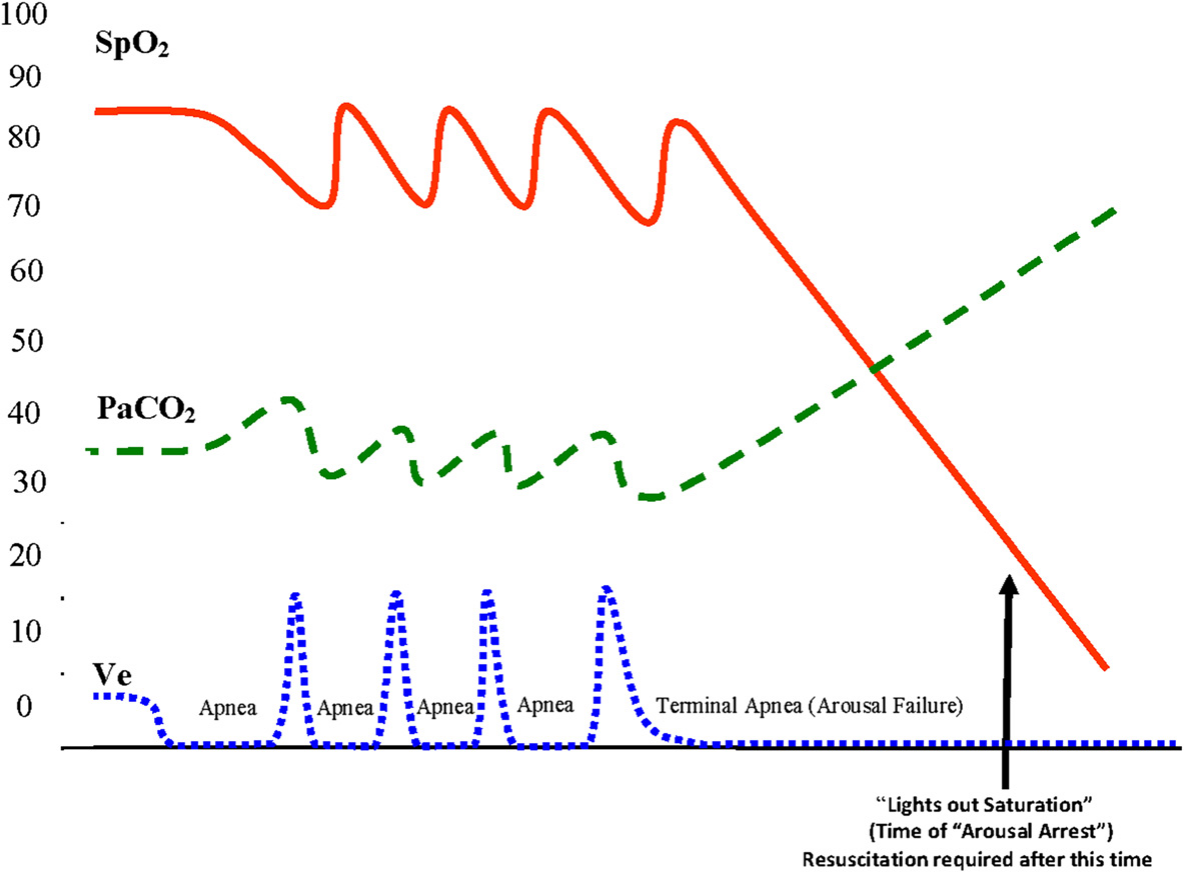
RECC Type III pattern of respiratory dysfunction (OSA).

The potential for such catastrophic outcomes and the enormous strain Obstructive Sleep Apnea (OSA) places on society with costly co-morbid consequences, have warranted several medical societies and foundations to recommend evidence based perioperative detection and management strategies. The Society of Anesthesiology and Sleep Medicine (SASM) has just released its detailed best practice consensus recommendations for managing perioperative OSA, and has provided this review article with its direct URL link to the information [64]. It can be argued that Type III is the most catastrophic of our three patterns because it is able to take an otherwise healthy patient’s life so suddenly (10 or less unobserved minutes) without any visible or audible warnings. This further supports those experts who insist that optimal GCF surveillance, when opioids are being utilized, must include some continuous electronic monitoring strategy capable of detecting Type III events [20,54]. Regarding sleep, the Type III event differs from Type II, which is induced by CO_2_ Narcosis, because it alone is a true sleep associated process. It starts as an arousal dependent sleep breathing disorder, where weak or incomplete arousal mechanisms fail completely from severe paroxysmal hypoxemia that induces an arousal arrest, and if left undiscovered an unwitnessed respiratory arrest [59-61]. Remember, Type II deaths are directly related to opioid induced respiratory depression and not disordered sleep breathing, although this issue becomes murky as our discussion on opioids in our next section will explain.The Type III pattern is not associated with elevated, upward trending sedation scores, which many clinical nurses place their absolute faith in regarding detection of all opioid associated threats. When awake, these patients can exhibit no pathognomonic symptoms or signs that give an impending Type III process away, including any evidence of sedation. In other words, patients with arousal failure are orphaned, hidden within typical perioperative populations. As shown in Figure 4 below, the sentinel instability components of this Type III pattern are the typical recurring cycles of obstructive sleep apneas in the presence of one final, complete arousal failure (arousal arrest).

**Figure 5:**
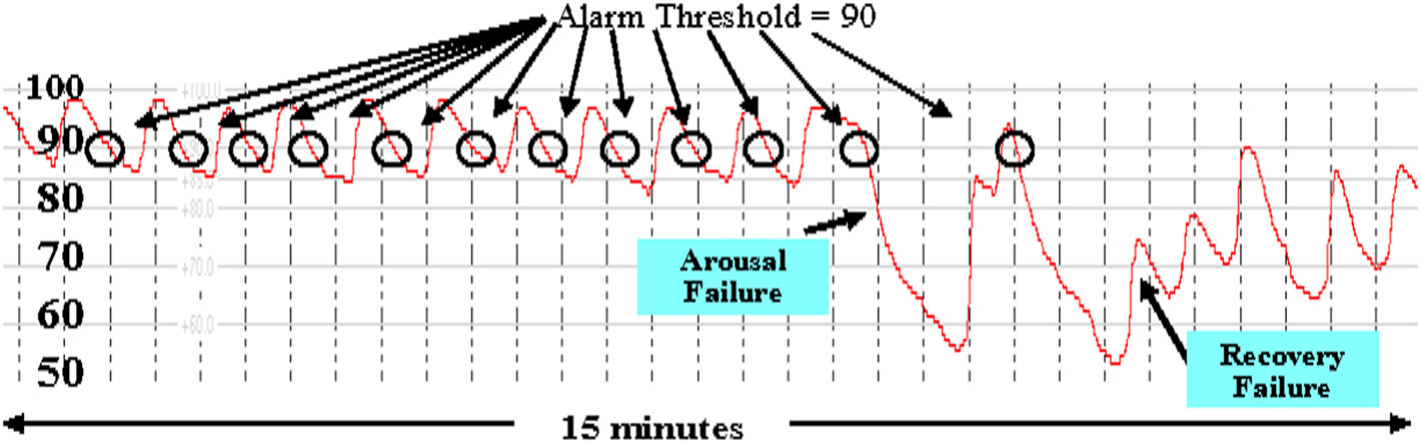
RECC type III pattern of respiratory dysfunction with arousal failure, recoveries, and alarm fatigue markers.

These Type III apnea patterns are comprised of repetitive reductions in airflow and SPO_2_ from sleep related cycling collapses of the upper airway [65,66]. This cycling (shown in Figure 5 below) with initial collapsing and then reopening of the upper airway, produces a typical, very distinct pattern of signal clusters that is reliably acquired through high resolution pulse oximetry (Note the potential for alarm fatigue if alarm threshold values are set near 90% SPO_2_). Interventions involving pressure support, e.g., CPAP and BiPAP, can diminish or completely resolve this Type III pattern as they do when used to treat RECC Type II *‘substitution*’ patterns and their associated risks.

Obstructive sleep apnea can be best understood as a condition where during sleep, one's upper airway collapses and is held closed by vigorous but ineffective respiratory effort. Each apneic event is generally terminated by a micro-arousal. The arousal then causes brief ‘overshoot’ hyperventilation that drives the PaCO_2_ below normal. The drop in PaCO_2_ triggers a fall in central ventilatory drive and upper airway tone. Since the upper airway is already unstable it collapses again, causing the cycle to reenter and self-propagate, producing its sentinel pattern of repetitive reductions in airflow and SPO_2_[65].

It has been theorized that chronic arousal failure may develop as a function of neural plasticity in response to repetitive exposures to rapid declines in oxygen saturation over many years. By the time these patients arrive for surgery, having been exposed to many years of repetitive desaturations during sleep, their arousal delays may have unknowingly progressed to now allow extreme levels of intermittent arterial hypoxemia. Their sleeping SPO_2_ values, already able to dive deeply during any given apnea, combine now with chronically reduced venous oxygenation supplies [58], expected postoperative reductions in functional residual capacity (FRC) [67], and ongoing intermittent additional ‘pilfering’ of oxygen content from the already compromised FRC due to recurring episodic apneas, to enable oxygen desaturation to now accelerate at rates over 1.5% per second [58]. This means that oxygen saturations (SPO_2_) can now dive to critical values with barely enough time for sufficient, contemporaneous hypercarbia to marshal a needed rescue arousal [68]. With even the slightest additional delay induced by drugs like opioids, an occasional patient’s arterial oxygen saturation will fall to the point where the brain no longer receives sufficient oxygen for central arousal to occur [4,5,46,59-61]. This is called the *‘Lights Out Saturation’* (LOS) because the human brain is incapable of generating sufficient anaerobic metabolism, depending solely on a continuous supply of oxygen and aerobic metabolism to support the generation of self rescuing arousals. It is as much a compromised oxygen reserve (FRC and venous) condition as it is a delayed neural arousal condition, each contributing to this infrequent, catastrophic, all-or-none self rescue failure. Because each sleep apnea ‘pilfers’ the FRC and venous supplies relentlessly, we like calling this FRC process ‘*bedside larceny*’.

Critical desaturation free falls become common even during relatively short apneas. These critical nadirs are hidden on traditional pulse oximeters because of their SPO_2_ averaging algorithms designed to smooth out sampling data and suppress nuisance alarms. Most often our patients tolerate this, even when opioids are generously provided. We may work long stretches, annoyed but falsely reassured by alarm breaches that consistently self correct…until an occasional disaster strikes! These few disasters are always catastrophic and perplexing to those lacking an OSA physiologic perspective.

Once SPO_2_ values fall below the ‘lights out’ critical value where the hemoglobin molecule simply cannot release sufficient oxygen, EEG slowing occurs promptly and arousal becomes totally suppressed [4,5]. When LOS is reached, airway reopening without resuscitation isn't to be expected. The body can remain alive for several minutes and in some instances even longer beyond the arousal arrest, continuing to burn glucose and fat as the heart continues to pump ever mounting CO_2_ stores throughout an anoxic body. If the patient is discovered at this later stage and resuscitated, immediately drawn blood gases could show PaCO_2_ to be moderately elevated, enough to disguise this Type III incident mistakenly as a Type II event. But unlike Type II events where an optimal continuous pulse oximetry ATV could be argued to be 90% SPO_2_, the preponderance of self correcting sleep apneas and associated SPO_2_ desaturations that are associated with the prevalence of OSA, opioid therapy, and postoperative conditions in general, make this alarm threshold value (ATV) for the Type III Pattern of Respiratory Dysfunction both unmanageable and unsafe on GCF when monitoring all patients without exclusion [4,5,7].

### The role of opioids

Not only are opioids capable of further delaying arousals in OSA patients already predisposed to having arousal failure [45,46,55], they can interfere with normal sleep architecture and have been associated with central apneas during sleep (CNS mediated apneas without any effort to breath) [69,70]. Unexpected central apneas have been observed even after opioids have been discontinued days earlier, possibly the result of REM sleep debt accumulated most often within the first 24 hours following surgery when opioid effects are most profound. This would argue for continuous monitoring to be extended beyond their discontinuation.

Also regarding sleep states, we can all appreciate how the experience of pain might impair sleep. But only recently has it been recognized that impaired sleep itself can directly exacerbate pain by causing hyperalgesia. This in turn, tends to solicit higher doses of opioids [71], again in turn perturbing sleep further and perhaps more importantly the ability of those with preexisting occult arousal failure and disordered sleep breathing to arouse from it. Studies on postoperative populations going as far back as 1984 [18,19] have appreciated a surprisingly high prevalence of postoperative episodic apneas, substantially beyond the expected prevalence attributable to OSA during the time those observations were made.

Perhaps most interesting and provocative are animal studies showing opioids to substantially disrupt the medulla’s regulation of breathing during sleep but not during wakefulness [72]. This research, published in 2011, demonstrated a critical site within the medulla of rats responsible for mediating opioid induced respiratory depression, called the preBotzinger complex. What’s thought provoking is that this complex appears to be the principal respiratory control center within the brain among many other scattered, non dominant control sites, and can appear to function perfectly normally when opioids are directly instilled into it during wakefulness. However, if these animals are then either exposed to anesthesia or allowed to sleep naturally, significant respiratory depression and fatal apneas begin to occur. Likewise, the preBotzinger complex was identified as wholly responsible for respiratory rate suppression following parenteral administration of opioids in these animals. The neurons responsible for this sensitivity express neurokinin-1 receptors that become selectively inhibited by opioids. However, most germane to our clinical interests, this raises an important question. Could our awake sedation scales be selectively sensitive and specific regarding some forms of opioid related respiratory dysfunction as we have already suggested (e.g. excellent sensitivity (few false negatives) and specificity (few false positives) with regard to the Type II pattern, but poor sensitivity regarding the Type III pattern where only a small dose of opioid without any sedative effect may be enough to critically delay an arousal dependent event once the patient falls asleep?

### Monitoring

One question should now be obvious. Why is it that we have not begun to continuously monitor every patient receiving parenteral (and neuraxial) opioids on our general care floors (GCF), especially while they sleep? The answer is both complex and revealing. GCF continuous pulse oximetry surveillance has been available and affordable for well over a decade, but the clarity we now have on this subject was earned from years of general care experience where nursing frustration was as evident as their confidence in the information gleaned from these new monitors. Valued hindsight, knowledge, and solutions eventuated, but progress has been slow, and much of it remained disconnected from the general care nursing domain. Unproven assumptions continue to remain fixed in place even now. For example, continuous pulse oximetry alarm threshold values (ATV) set at or near 90% SPO_2_ for the GCF have never been proven in any clinical trials to be optimal for this particular environment. History suggests this ATV was adopted simply because of the positive experiences encountered in earlier years when it was recommended through expert consensus for use in the OR, PACU, and Critical Care environments. GCF nurses, once faced with having to fit continuous pulse oximetry surveillance using a 90% SPO_2_ ATV into their everyday work flow, learned quickly that certain unsupportive behaviors predictably followed because of the inevitable alarm fatigue. These behaviors ranged from developing patient monitoring exclusion policy or simply ignoring the vast numbers of self correcting alarms [7], to outright monitor abandonment. The behaviors likely evolved as do self initiated reorganization efforts in times of unexpected chaotic conditions or emergency, and were amazingly consistent across the nation. Nellcor and Masimo, two major pulse oximetry leaders during this time, competed fiercely with one another to optimize signal clarity and reduce unnecessary noise, but GCF oximetry adoption in the late 1990s preceded our general appreciation for the prevalence and physiologic challenges later attributed to Obstructive Sleep Apnea (OSA), especially in our postoperative patient populations. Our specialty societies did not begin aggressively addressing these OSA issues until well into the 2000s, long after GCF nurses had already fomented their own ‘counter’ monitoring policies to accommodate an otherwise cacophony of alarm noise being caused by countless self correcting 90% SPO_2_ breaches from sleep apnea [7,51]. Likewise, threshold decision making and expert consensus on a variety of thresholds [10-13] in the 1990s and 2000s was widely excepted by many of medicine’s most respected thought leaders. A variety of unproven physiologic thresholds had been assigned in 1991 and again in 2003 to comprise the fundamental definition of our leading cause of death in hospitalized patients today, sepsis [11,12]. Our nursing colleagues were to find little support in those early GCF oximetry years to challenge the validity of this single 90% alarm threshold value. In hospitals where we had actually interfaced with the general care nursing leadership routinely, our experiences had been consistent. The 90% SPO_2_ pulse oximetry ATV was interpreted to be a standard of practice and believed to be deemed as such by expert consensus, regardless that such ‘consensus’ had never anticipated oximetry’s use on general care floors. Any significant change to this ‘standard of practice’ would have to be proven through clinical trial to be superior, regardless that the ‘expert consensus’ originating this arbitrary belief had never met this benchmark. So rather than regarding alarm threshold values (ATV) as flexible tools best custom designed to fit the needs of the patients they serve, this 90% SPO_2_ ATV calcified in unchanging nursing policy for years, and remains today still the GCF ATV of choice in many institutions across the nation. Current controversy regarding the non reproducibility of clinical trials and our lack of therapeutic progress over the last two decades regarding sepsis, other than supportive care improvement [14-17], are forcing our sepsis experts to reconsider if unproven biomarker thresholds can indeed ever serve as reliable diagnostic and defining standards, and if any unproven expert consensus based decision should ever be allowed to rise to Level 1 clinical evidence equivalency. This controversy may hopefully reward us all with the proper balance of skepticism and zeal to make more prudent decisions regarding patient care going forward.

Useful knowledge continues to emerge regardless our penchant for human folly. Sleep medicine continues to be somewhat removed from those who deliver clinical care on the GCF, although the Society of Anesthesia and Sleep Medicine (SASM) has now begun making inroads with consensus recommendations for best practices based on Level 1 evidence [64]. Despite consensus recommendations from the Anesthesia Patient Safety Foundation (APSF) advocating twice in the last 8 years (2006 and 2011) for all perioperative GCF patients receiving opioids to be monitored with some form of continuous electronic surveillance, only two institutions have trialed and published their successes [7,73], with only one using a single monitor to accomplish this. The APSF has provided us with a direct URL link to its consensus recommendations for best practices regarding continuous electronic monitoring [54].

Now that you’ve become very familiar with the information essential to understanding the three pattern types of respiratory dysfunction that place patients at risk on hospital GCF, you should realize as well our challenge is to figure out how best to monitor all GCF patients for all three patterns simultaneously and continuously using preferably just one electronic surveillance technology and one alarm threshold value to do it. The good news is that this has actually been done to a great extent already using continuous pulse oximetry, the monitor we’ve had the most experience with on GCF, with excellent outcomes to show for this applied innovative strategy. However, this strategy was created prior to and without benefit from our three pattern cognitive framework creation that clarifies exactly why any one pulse oximetry alarm threshold value can not optimally service all three pattern types simultaneously. This actually presents us with an opportunity to create a non onerous, new strategy that combines the excellent work done at Dartmouth (*to be detailed momentarily*) with our cognitive framework to assure all GCF patient populations optimal safety.

Referring to our three pattern cognitive framework, we should all realize now that optimal early detection of the most prevalent of our three RECC patterns (Type I) requires only a knowledgeable clinical nurse who will listen and respond to all early complaints of shortness of breath. Any downward SPO_2_ changes coincidentally discovered with these ‘early’ complaints do yield valid information, but reflect only that the Type I RECC processes are no longer in their early stages because the benefits of compensatory respiratory alkalosis through hyperventilation no longer mask the advancing PaO_2_ reductions (FRC *replacement*). There is no optimal oximetric threshold value that can alarm us to take proper early action with Type I events. Our patients’ safety depends solely on the astute judgment of our nurses who are most often the first clinicians made aware there’s a problem. Any further delays should be avoided and certainly any breach of our customary 90% SPO_2_ ATV generally represents a significant detection delay with morbidity and mortality to be expected.

Early detection of the less common, but more catastrophic RECC Type II pattern likewise requires a knowledgeable, observant nurse. While there are occasional clinicians who argue that GCF nurses can provide a safe environment without continuous electronic monitoring with regard to both Type I and Type II events, we strongly believe this isn’t the case. We, along with the Anesthesia Patient Safety Foundation and many other leading experts [6,20,31-34,54], believe the plethora of competing nursing responsibilities that encroach on nursing time along with the unpredictability of GCF patient populations, especially at night where sleep can so easily mask CO_2_ Narcosis, make the risk that accompanies intermittent monitoring untenable. Unlike the smooth averages generally relied on to design nursing GCF coverage, the actual minute to minute workflow is far more unpredictable and clumpy with mini crisis the rule rather than exception. Additionally, optimal mindsets for preempting the unexpected (e.g. detecting early RECC) are to believe everything is wrong until proven otherwise. Maintaining this mindset on busy hospital GCF is not only difficult, it’s culturally foreign [74]. Caregivers predisposed to assuming things are wrong until proven otherwise (known as *sense-makers*[74]), gravitate toward jobs in hospital EDs, ICUs, PACUs, and ORs. The general care floors have always had to contend with an underlying presumption that their patients are stable. GCF culture is referred to as *decision-making*[74], where the patient to nurse ratios and work pace encourage all to be assumed right until proven otherwise*.* Even in hospital environments where sense-making is expected to predominate (e.g. PACU), Type II events occasionally get discovered quite late with skilled nurses standing literally at the bedside providing one-on-one care, so convincingly the Type II pattern is able to mimic restorative sleep [34].

When continuous pulse oximetry is used for surveillance on these patients breathing either room air or air supplemented with the low flows of O_2_ that deliver a FIO_2_ less than .28 (see Table 1), any downward trend in SPO_2_ values can reliably warn knowledgeable nurses of early evolving Type II patterns and an FRC ‘*substitution*’ process well before a 90% SPO_2_ threshold alarm can give it away. Because 90% SPO_2_ ATV function under these conditions more as safety net tools than an optimal early detection strategy, learning to respond to these SPO_2_ trends while utilizing awake sedation scoring, and appreciating the physiologic masking that accompanies supplemental oxygen delivery at FIO_2_ greater than .27, becomes the most reliable early detection strategy currently available for Type II events. Simply waiting on an oximetry alarm to sound regardless where the alarm threshold value is set (both for Type I and II events) suggests issues regarding competence and should be discouraged. Once a Type II pattern is suspected, nurses should never hesitate to arouse these ‘sleeping’ patients, but first should spend a moment observing their respiratory rates and depths of breathing to gain experience and realize how misleading these observations can sometimes be.

**Table 1:** **Simulated SPO**_**2 **_**values associated with FIO**_**2 **_**and PaCO**_**2**_**/arterial pH**

**FIO**_**2**_	**PaCO**_**2 **_**55 mmHg (pH 7.26)**	**Oximeter 90% alarm breach**	**SPO**_**2 **_**drift**	**PaCO**_**2 **_**70 mmHg (CO**_**2 **_**Narcosis)**	**Oximeter 90% alarm breach**	**SPO**_**2 **_**drift**
**30 yo Patient Model**						
.21	SPO_2_ 91%	-	+		+	+
.24	SPO_2_ 95%	-	-	SPO_2_ 89%	+	+
.27		-	-	SPO_2_ 93%	-	+
.30	SPO_2_ 98%	-	-	SPO_2_ 95%	-	-
**50 yo Patient Model**						
.21	SPO_2_ 89%	+	+		+	+
.24	SPO_2_ 94%	-	+	SPO_2_ 87%	+	+
.27		-	-	SPO_2_ 93%	-	+
.30		-	-	SPO_2_ 95%	-	-
**75 yo Patient Model**						
.21	SPO_2_ 87%	+	+		+	+
.24	SPO_2_ 93%	-	+	SPO_2_ 84%	+	+
.27	SPO_2_ 96%	-	-	SPO_2_ 91%	-	+
.30		-	-	SPO_2_ 95%	-	-

The RECC Type III pattern (FRC ‘*bedside larceny*’) challenges us very differently. While early Type I event detection does not rely on continuous electronic monitoring at all and early Type II events do but rely on SPO_2_ signal trending, not arbitrary safety net alarm values considered most popular in any given individual institutions, e.g., SPO_2_ 90%, there is currently no other way to reliably detect Type III events today that come on so suddenly and take lives so quickly, than by deploying a safety net oximetry threshold alarm. Here, choosing a threshold value that is both proven capable of permitting universal population surveillance and being capable as a safety net for all three patterns becomes essential. What has prevented most of us from accomplishing this have been the historic issues discussed earlier that led to pulse oximetry’s GCF association with unrelenting alarm fatigue [14,15]. Now enlightened, we should begin to again make appropriate use of new alarm threshold values as helpful clinical tools whenever they have been shown to reliably serve the patients for whom we care, e.g., the 90% SPO_2_ ATV used in the OR and Critical Care Units, and now the Dartmouth experience on GCF.

### An example of successful monitoring strategies at Dartmouth

In 2007 Dartmouth-Hitchcock Medical Center initiated a program called the Patient Surveillance System (PSS) on a 36 bed postoperative orthopedic unit. PSS required all its patients to be electronically monitored with continuous pulse oximetry, and all threshold breaches to be transmitted electronically through pager devices to the caregivers in charge. The program was tightly aligned with their Hitchcock Early Response Team (HERT), a Rapid Response program that brings critical care expertise to the bedside once RECC are detected [7,51].

This trial began as a Before-and-After Concurrence Study with data analyzed before and after intervention for the PSS unit and compared with two other units that care for surgical patients. All comparative data was collected concurrently. Data were collected prospectively hospital wide. No change of data collection was performed during the study period. Data included incidence of emergent airway rescues, code blue and HERT activations, transfer to the intensive care unit, death, patient demographics, patient diagnosis related group, length of stay, and patient satisfaction with pain control. For comparison purposes, rescue events were tracked as per 1,000 discharges (as done by the Institute for Healthcare Improvement) for the PSS and comparison units. Transfers to the ICU were tracked as transfers per 1,000 patients days for all units (as the most commonly used denominator for patient transfers).

Prior to beginning the study, a month was spent observing the physiologic responses of the PSS patient population in order to design an ‘optimal’ ATV that trades off earlier notification of some deterioration against limiting the nuisance alarms caused by self correcting changes or false readings. It was discovered that patients spent 13% of their time at < 93% SPO_2_ and 6% of their time < 90%, both generating several false alarms per patient per hour. This prevalence of nuisance alarms was observed to have desensitized staff, leading to delayed response and in one trial run to where nurses simply began ignoring many of the alarm alerts. To reach a balance between actionable and false positive alarms in this work, the following alarm thresholds were chosen: SPO_2_ less than 80% and heart rate less than 50 and more than 140 beats per minute. Notification delay was the other important concept in alarm frequency management. Appropriate delay was thought to eliminate many transient and motion artifact-generated false alarms. A 15 second audio alarm delay at the bedside and an additional 15 second delay for pager annunciation, leading to a 30 second total delay before a nurse would be notified by pager of violation of alarm thresholds was instituted. This was the first published report of universal surveillance (100% monitoring of patients during their entire hospitalization when not directly observed by the healthcare team) rather than condition monitoring in postoperative clinical practice. It was a new approach to detect unrecognized postoperative deterioration and a departure from the concept of optimized individual care to optimized population care made necessary because of the documented failure of successfully identifying patients at risk for adverse events [20], a significant precursor in mortality and morbidity for GCF hospital patients [75].

Results from this PSS study demonstrated first a very high patient acceptance rate for the continuous pulse oximetry monitoring compared with their earlier trials using modalities such as end tital CO_2_ nasal cannulas: 98.2% (only 1.8% refusing to continuously wear a pulse oximetry sensor because of inconvenience). Likewise system uptime was extraordinarily reliable: 99.9995%. The number of alarms averaged four per patient per day or two per 12 hour nursing shift. Observed deaths after implementation were 2 as opposed to 4 in the previous time frame. Length of stays were 3.69 and 3.68 days for all patients before and after implementation, and 3.29 before and 3.20 days after for patients who did not have ICU transfers associated with their care. Rescue events in the PSS decreased from 3.4 to 1.2 per 1000 patient discharges (*P* = 0.01). In this 36 bed unit, this means an effect size change from 37 to 11 rescue events annualized. ICU transfers declined from 5.6 to 2.9 per 1000 patient days, and over one year this equated to a decrease from 54 to 28 transfers. They concluded that the results demonstrated that universal continuous pulse oximetry surveillance could improve outcomes on a postoperative orthopedic ward setting and that these gains may hold true for other postoperative settings as well.

Fast forwarding to today, all medical and surgical patients at Dartmouth have been mandated since 2010 to be continuously monitored with continuous pulse oximetry when they are not being directly observed by a health care provider. Expansion of this patient surveillance to other units have had significant positive effects on outcomes on all surgical units, just not on the medical units with a low prevalence for adverse events [51]. Significant reductions were seen in rescue events (0-65%) and in ICU transfers (0-50%) with greater reductions on wards with higher utilization of the system, greater baseline risks, and higher opioid consumption. Use of opioids and number of opioid reversals have not changed. However, no patients have suffered irreversible severe brain damage or died as a result of respiratory depression from opioids since PSS was instituted on the original study unit in December of 2007. Also noted was that on surgical units, opioid consumption is greater than on medical units, and the majority of rescue events (> 75%) are respiratory in nature. Cost effectiveness was appreciated as well stemming primarily from cost savings due to decreased ICU transfer rates. Yet despite these remarkable outcome gains, those in charge of the PSS program today readily admit that their patients still have adverse events requiring interventions and escalations of care. Its chosen ATV has served very well as a safety net for their entire patient GCF populations, but additional tools or education will be needed to optimize these advances by making the adverse events easily detectable in their earliest phases.

## Conclusion

The Dartmouth experience [51], when examined through the lens of our RECC three pattern cognitive framework, offers the solution to our challenge set forth to figure out how best to surveil all GCF patients for all three patterns simultaneously and continuously using just one electronic surveillance technology and one alarm threshold value to do it. This experience is the only collection of GCF studies completed that prove there is a continuous pulse oximetry SPO_2_ ATV strategy capable of providing a safety net for all unexpected clinical deterioration synonymous with our RECC patterns. The proof is reflected in its significant patient outcome improvements and absent mortality and morbidity for serious opioid associated events since inception. While Dartmouth PSS leadership acknowledges that an 80% SPO_2_ ATV with additional 30 second alarm delay is still a trade off sacrificing earlier notification of some clinical deterioration against limiting the nuisance alarms caused by self correcting changes, our cognitive framework clarifies the precise nature of this clinical ‘deterioration’. In doing so, we have been able to devise non onerous educational recommendations regarding how to utilize information early from patients themselves and continuous pulse oximeters other than their capacity to alarm, for optimal early detection of Type I and Type II RECC events. Type III events are managed best with exactly what the Dartmouth experience currently uses for its PSS alarm strategy. Additionally, the original PSS study [7] discusses Dartmouth’s own failures and reasons for them when attempting to use ATV at 90% SPO_2_. Those many institutions still clinging to 90% SPO_2_ ATV for GCF use, no longer can justify this based on standard of practice or performance.

Once again, RECC Type I Patterns of Respiratory Dysfunction can be thought of as FRC ‘*replacement*’ processes, and should be aggressively evaluated at the first complaints of patients stating they are short of breath. The RECC Type II Pattern of Respiratory Dysfunction can be thought of as an FRC ‘*substitution*’ process, easily trended by noticing downward SPO_2_ trends on a continuous pulse oximeter provided you are knowledgeable and aware of the respiratory masking issues from supplemental oxygen provided at flow rates above FIO_2_ .27. (Refer to Table 1) The RECC Type III Pattern of Respiratory Dysfunction involves delays in arousal and depletion (*bedside larceny*) of the body’s oxygen reserves (FRC and venous O_2_) to mount its terminal apnea with arousal arrest, culminating in an immediate full respiratory arrest if left undetected. Integrating the Dartmouth PSS monitoring experience with your new understandings of our three RECC patterns should provide your postoperative patients going forward with the safety they expect and deserve.

## Abbreviations

ATV: Alarm threshold values; FIO_2_: Fraction of inspired oxygen; FRC: Functional residual capacity; GCF: General care floors; OSA: Obstructive sleep apnea; ODC: Oxyhemoglobin dissociation curve; RECC: Rapidly evolving clinical cascades; SPO_2_: Arterial oxygen saturation.

## Competing interests

JPC is a consultant for Lyntek Medical Technologies, contracted to write detailed articles based on his own deep knowledge and understanding for the subject of pattern recognition and the current state of hospital monitoring and safety. He holds no equity position in Lyntek Medical Technologies and has no other competing interests. CRJ has no competing interests.

## Authors’ contributions

Both authors contributed to the drafting and revisions that produced this final work. Each contributed to the intellectual content, bringing their deep knowledge and experience to the final conceptualization that comprises this review. Both authors read and approved the final manuscript.

## Authors’ information

JPC has been an Emergency Medicine Physician, an Anesthesiologist, and past Chief of Staff of Hoag Memorial Hospital Presbyterian, Newport Beach, CA. He founded and is clinical advisor for the Hoag Rapid Response Initiative, has been a past member of clinical advisory boards for two leading oximetry companies, and has been an Assistant Clinical Professor in the Department of Anesthesiology, UCLA David Geffen School of Medicine. He currently consults for Lyntek Medical Technologies and serves the Clinical Committee of the Society of Anesthesia and Sleep Medicine.

CRJ is an Assistant Professor at the University at Buffalo and Nurse practitioner at the Thompson Health Sleep Disorders Center. She is co author on the ASPMN guidelines for monitoring patients for opioid induced respiratory depression and served on the panel of experts to develop a quality measure for monitoring hospitalized patients. Her research program aims to promote safe and effective pain management.
